# Neurobiological Risk Factors for the Development of Internet Addiction in Adolescents

**DOI:** 10.3390/bs9060062

**Published:** 2019-06-14

**Authors:** Sergey Tereshchenko, Edward Kasparov

**Affiliations:** Research Institute of Medical Problems of the North, Federal Research Center “Krasnoyarsk Science Center of the Siberian Branch of the Russian Academy of Sciences”, Krasnoyarsk 660022, Russia; impn@impn.ru

**Keywords:** Internet addiction, adolescents, comorbidity, neurobiology, neuroimaging, neurotransmitters, gene polymorphism

## Abstract

The sudden appearance and spread of Internet addiction in adolescent populations, in association with the rapid escalation of consumed Internet content and the broad availability of smartphones and tablets with Internet access, is posing a new challenge for classical addictology which requires urgent solutions. Like the majority of other psychopathological conditions, pathological Internet addiction depends upon a group of multifactor polygenic conditions. For each specific case, there is a unique combination of inherited characteristics (nervous tissue structure, secretion, degradation, and reception of neuromediators), and many are extra-environment factors (family-related, social, and ethnic-cultural). One of the main challenges in the development of the bio-psychosocial model of Internet addiction is to determine which genes and neuromediators are responsible for increased addiction susceptibility. This information will herald the start of a search for new therapeutic targets and the development of early prevention strategies, including the assessment of genetic risk levels. This review summarizes the literature and currently available knowledge related to neurobiological risk factors regarding Internet addiction in adolescents. Genetic, neurochemical and neuroimaging data are presented with links to actual pathogenetic hypotheses according to the bio-psychosocial model of IA forming.

## 1. Introduction

The explosive growth of the Internet usage in our day-to-day life has created numerous technological advantages. Simultaneously, it has had a range of side effects impacting psychological and somatic health, which are especially important for a growing body and unformed mental functions. Internet addiction (IA) is a relatively new psychological phenomenon, most commonly marked in socially vulnerable groups (e.g., in adolescents and young adults). IA is one of the 11 forms of addictive behavior. At present, it has suggested diagnostic criteria that allow the framing of the pathological component of addiction with its signs of psychological disturbances. Internet gaming disorder is included in the Diagnostic and Statistical Manual of mental disorders, fifth edition (DSM-V), but is placed in a separate chapter titled “Conditions for further study”. “Predominantly online gaming disorder” is planned as a separate entity in the International Classification of Diseases (ICD-11) [1].

In terms of classical psychology and psychiatry, IA is a relatively new phenomenon. The literature uses interchangeable references such as “compulsive Internet use”, “problematic Internet use”, “pathological Internet use”, and “Internet addiction”.

From the moment when the IA phenomenon was first described in the scientific literature [2,3,4] until now, discussions about the exact definition of this psychopathological condition are ongoing [5,6]. Psychologist Mark Griffiths, one of the widely recognized authorities in the sphere of addictive behavior, is the author of the most frequently quoted definition: “Internet addiction is a non-chemical behavioral addiction, which involves human-machine (computer-Internet) interaction” [7].

Even though the common definition and diagnostic criteria of IA are continuously under debate, psychologists and psychiatrists have agreed on the four components essential to this diagnosis [8,9].

(1) Excessive Internet use (especially when characterized by loss of time or neglecting basic functions): compulsive striving for Internet usage, growing importance of Internet in an adolescent’s system of personal values;
(2) Withdrawal symptoms: mood swings (abstinence withdrawal symptom) when Internet is unavailable (anger, depression, and anxiety);
(3) Tolerance: need to spend increasing amounts of time on the Internet, exemplified by the need for increased use of the Internet to relieve negative emotional symptoms; and
(4) Negative consequences: excessive engagement in Internet use, contrary to negative psychosocial outcomes; loss of previous hobbies and entertainments as a result of such engagement; loss of social relations, educational, and sport opportunities resulted from undue usage of Internet; quarrels and lies with regards to using the Internet; relapse: self-control failure in relation to Internet use.

Currently, several etiopathogenetic models have been proposed for IA formation in adolescents [10]. Some researchers attribute the predisposition of adolescents to the IA onset with the lack of effective effortful control, high impulsivity, and a highly activated reward circuitry, which is largely due to incomplete neurobiological maturation of adolescent’s brain [11,12]. Other authors propose a “component bio-psychosocial model” combining psychosocial factors or problems—in particular, relational problems with peers and/or with adults—with the intergenerational transmission of psychopathological [10]) and neurobiological risk factors for the development of IA [13,14]. Some of the neurobiological risk factors for the development of IA in adolescents in accordance with the bio-psychosocial model will be discussed in this narrative review.

## 2. Epidemiology of Internet Addiction

In population-based investigations, IA criteria presence must be verified by psychological questionnaires that have been specially designed and validated for adolescents. The first questionnaire, aimed at IA verification, is the Kimberly Young Internet Addiction Test, which was validated in 1998; it was developed to identify Internet addiction. Young’s pioneering research played a significant role in IA diagnostics using standardized means. Since then, a range of new questionnaires has appeared, matching the modern development of clinical and adolescent psychology to a greater extent. The Chen Internet Addiction Scale (CIAS) is among them [15], developed especially for adolescents.

The data from the international literature on IA in adolescents indicates a prevalence within the range of 1% to 18% [6], depending on ethnic social groups and the diagnostic criteria and questionnaires used in the study. In Europe, the IA prevalence in adolescents is 1–11%, with an average of 4.4% [16]. In the USA, the IA prevalence in adults is 0.3–8.1% [17]. Adolescents and young adults in Asian countries (China, South Korea, and others) show a considerably higher IA prevalence of 8.1–26.5% [18,19]. In Moscow, Russia, Malygin et al. tested 190 schoolchildren of grades 9–11 (aged 15–18 years). Their research found that 42.0% of adolescents showed excessive Internet use (pre-addictive stage, according to the author’s definition) and 11.0% had manifested signs of IA. In this study, the Russian version of the CIAS questionnaire, validated by the authors, was used [20]. In another study conducted in Russian adolescents, the authors found that among 1,084 adolescents with an average age of 15.56 years, 4.25% had IA as a diagnosis and 29.33% showed excessive Internet use (pre-addictive stage, according to the author’s definition) [21]. 

## 3. Comorbidity of Internet Addiction

Numerous studies have convincingly demonstrated IA comorbidity with a broad range of psychopathologic conditions. Ho et al. in their meta-analysis demonstrate IA comorbidity with depression (OR = 2.77, CI = 2.04–3.75), anxiety disorders (OR = 2.70, CI = 1.46–4.97), attention deficit–hyperactivity disorder (ADHD); OR = 2.85, CI = 2.15–3.77) [22]. In their systematic review, Carli et al. showed that depressive disorders and ADHD have the strongest association with IA. A lesser but still meaningful association was found with anxiety, obsessive compulsive disorders, social phobia, and aggressive behavior [23]. The same conclusions were supported by another systematic review [24]. Durkee et al.’s [25] research involved a representative sample of 11,356 adolescents from 11 European countries and found that IA is associated with self-destructive and suicidal behavior as well as depression and anxiety. The same results were obtained by Jiang et al. [26]. Other investigators proposed that IA is associated with definite personal features, namely “sensation seeking”. This is frequently described by Western authors as a striving for new, unordinary, and complicated sensations, which are often risky [27]. In their longitudinal study, Guillot et al. demonstrated IA associations with anhedonia in adults (i.e., weakened ability to feel pleasure, which is typical for depressive disorders) [28].

IA associations with psychosomatic diseases are not clear, though they might be possible given that comorbid factors may be mutually connected (e.g., anxiety, depressive, and obsessive- compulsive disorders). Wei et al. found that IA is associated with chronic pain syndromes [29]. Cerutti et al. found no statistically meaningful associations between IA and tension headaches/migraines, although somatic pain symptoms, in general, were frequently found in IA patients [30]. Other authors found an association of IA with sleep disorders in adolescents [31]. Similar data have been reported for a sample of Japanese schoolchildren [32].

## 4. Pathogenesis of Internet Addiction in Terms of Neurobiology

The development of the brain during the adolescence is characterized by the formation pathways in the limbic system and prefrontal cortical regions at different periods of time [33]. In adolescents, an extended prefrontal cortex development time compared to that of the limbic system results in weakened inhibition from the side of the cortical regions toward underlying subcortical structures, resulting in more prominent impulsivity, which contributes to high-risk behavior [34].

Thus far, numerous studies have been conducted to study Internet addiction pathogenesis using different neurovisualization methods, including different variants of brain structural magnetic resonance tomography (e.g., voxel-based morphometry, diffusion tensor imaging, and functional magnetic resonance imaging) and nuclear magnetic resonance tomography (e.g., positron emission tomography and single photon emission computed tomography). Based on the listed methods, the following IA-associated structural transformations in the brain have been detected [35,36,37]: lowered density of gray matter in different regions, including the prefrontal, orbitofrontal cortex, and supplementary motor area [38]; abnormal functional activity of brain regions associated with the reliance on rewards [11]; activation of sensory motor synchronization with simultaneous lowering of audiovisual synchronization [39]; activation of brain regions related to the formation of uncontrollable desires and impulsivity; glucose-increased metabolism in brain regions associated with impulsivity; dependence on reward and aspiration for the repetition of the experienced somatic sensations [40]; and dopamine-enhanced secretion with further lowering of dopamine receptor availability in the striatal region [41]. The analysis of the electric encephalogram event-related potentials showed a decreased time of response, which may be associated with the disturbance of voluntary regulation [42].

A whole range of neuromediators may be involved in the neurobiological mechanisms of IA formation in adolescents. For instance, oxytocin—the hormone of trust, social connections, and emotional attachment bonds—plays a vitally important role in establishing direct social emotional contacts in adolescents’ environments. Numerous studies have demonstrated associative bonds between different polymorphic regions of the oxytocin receptor and the *CD38* gene in various psychiatric and neurodevelopmental disorders, including autistic spectrum disorders. This was analyzed in detail in the review by Feldman et al. [43]. Oxytocin concentrations in saliva were found to be negatively correlated with the expressiveness of behavioral problems, which were identified using the Strengths and Difficulties Questionnaire [44]. The same authors specified that oxytocin production is decreased in children with callous and unemotional traits. Sasaki et al. did not find any associations between oxytocin concentration in saliva and the expressiveness of depression symptoms in adolescents, though patients with treatment-resistant depression showed higher levels of oxytocin than the control cohort with non-resistant depression [45]. The oxytocin plasma level was decreased in children with attention deficit/hyperactivity syndrome, and it was negatively correlated to impulsivity and inattentiveness [46,47].

Many studies have reported a pathophysiological connection between the oxytocinergic system and the formation of different forms of addictive behavior in adolescents and young adults [48]. The efficacy of oxytocin administered in therapy for different types of addiction (especially alcoholism) was demonstrated both using animal experimentation [49] and clinical research [48]. The main mechanisms of oxytocin therapy in chemical addictions are the alleviation of physical symptoms and an increase in the emotional tonus in abstinence, lower anxiety, growth of perceptivity to verbal intervention, easier renewal of social contacts, and the physiologic reduction of the stated tolerance. Since psychological stress is an important etiological cause of the formation of pathological addictions, the hypothesis about the oxytocin anti-stress effect as the possible protection factor appears convincing [50]. Oxytocin anti-stress influence was realized through the inhibition of excessive stressor activation of the hypothalamic-pituitary-adrenal axis, regulation of the mesolimbic dopamine system of reward, and the production of the corticotropin-releasing hormone.

The possibility of a genetically-determined predisposition to addictive behavior was revealed. This predisposition was found to be associated with the inadequate efficiency of the oxytocinergic system. Thus, genetic tests for 593 adolescents aged 15 years resulted in finding the association between frequent alcohol drinking and the formation of alcohol addiction in boys (not in girls) up to the age of 25 with homozygosity related to the A allele variant of the rs53576 polymorphic region of the oxytocin receptor gene [51]. An association between adolescent suicidal behavior and this homozygosis variant of the *OXTR* gene was reported by Parris et al. [52]. 

The contribution of the following listed substances in the pathogenesis of adolescent addictive behavior is highly likely, but has not yet been studied well. In addition to oxytocin, there are the following perspective neuromediators:

(1) Melanocortin (α-Melanocyte-stimulating hormone (α-MSH)): Orellana et al. [53] proposed the important role of melanocortin in the formation of pathologic addictions in adolescents.
(2) Neurotensin: Neurotensin is actively involved in the modulation of dopamine signaling and the formation of pathological addictions; there are cases of the successful treatment of some forms of addiction with synthetic neurotensin [54].
(3) Orexin: Orexin may be involved in the formation of disturbed sleep and the formation of addictive behavior [55].
(4) Substance P (neurokinin A): A disturbance in the production of substance P is thought to be related to the formation of many forms of pathological addictions; at present, there are trials underway testing the efficacy of neurokinin receptor activity modulation in therapy for addiction [56,57].

## 5. Genetics of Internet Addiction

In contrast to he other forms of addictive behavior (like gambling and psychoactive substance abuse), little research has focused on the genetic predictors of Internet addiction. For example, in the first twin study conducted in 2014, the authors examined 825 Chinese adolescents and showed association with inherited component in 58–66% of the population [58]. Later, the researchers of twin cohorts from the Netherlands (48% in 2016 [59]), Australia (41% in 2016 [60]), and Germany (21–44% in 2017 [61]) came to similar conclusions. Therefore, the presence of a genetic component in IA formation was credibly supported by twin studies for different populations. However, specific genes that might be associated with inheritance mechanisms have not yet been identified. Four pilot research studies verified the polymorph regions of five candidate genes:

(1) rs1800497 (dopamine D2 receptor gene (*DRD2*), Taq1A1 allele) and rs4680 (methionine variant of dopamine degradation enzyme catecholamine-o-methyltransferase (*COMT*) gene): The first of these studies concentrated on adolescents in South Korea. The study demonstrated that the bond of minor alleles is associated with dopamine low production (rs4680) and low number of dopamine receptors in prefrontal cortex (rs1800497) in the presence of a pathological obsession to Internet games [62]. The mentioned allele variants can be simultaneously associated with the predisposition to alcoholism, gambling, and ADHD.
(2) rs25531 (serotonin transporter gene (*SS-5HTTLPR*), short allelic variants): Lee et al. [63] showed that the short allele variants of the serotonin transporter gene can be associated with pathological Internet addiction. As was supported by numerous studies, the said genetic variants were also associated with a predisposition to depression—the most prevalent comorbid disorder in Internet-addictive subjects [64].
(3) rs1044396 (nicotinic acetylcholine receptor subunit alpha 4 (*CHRNA4*) gene): a small sized case-control study by Montag et al. [65] showed the presence of an association with the CC genotype of the polymorphism rs1044396, which is also related to nicotine addiction and attention disturbances.
(4) rs2229910 (neurotrophic tyrosine kinase receptor type 3 (*NTRK3*) gene): A pilot study by Jeong et al. [66] was aimed at a specific exome and involved 30 adults with Internet addiction and 30 healthy subjects. The research included studying 83 polymorph regions and revealed statistically convincing associations with only one region: rs2229910. Presumably, this is associated with anxiety and depressive disorders, obsessive-compulsive disorders, and psychologically-determined nutrition diseases.

The prevalence of some polymorphic regions supposedly associated with the formation of Internet addiction can have statistically meaningful distinctions in different ethnic groups. The analysis of the available scientific literature shows that the ethnic factor in the search of these genetic associations has been given insufficient attention. The systematic review by Luczak et al. [67] concentrated on the ethnic peculiarities of the 11 forms of addictive behavior. Only one study was found (quoted previously in the review by Kuss et al. [16]) where the IA ethnic factor was considered [68]. The authors examined 1470 college students with compatible social cultural living conditions. They revealed a high frequency of IA in representatives Asian (8.6%) in comparison with non-Asian (3.8%) nationalities. The same review quotes a range of scientific sources, revealing the high prevalence of computer game dependence in non-European Americans (e.g., native Americans and black Americans) compared to Caucasian (white) ethnicities [67]. In a large multicenter (11 countries) trial focused on European Internet-addicted adolescents, the authors had found it to be the most expressed comorbidity with suicidal behavior, depression, and anxiety, but the contribution of each of comorbidity was different in each country. The authors concluded that further research was necessary with the obligatory consideration of social, cultural, and, probably, ethnic (genetic) characteristics [25,69]. From our point of view, the analysis of ethnic and geographic distinctions related to Internet addiction, which simultaneously accounts for ethnic peculiarities in the prevalence of genotype distinctions of the populations, is a promising area for modern neurogenetics with regards to adolescent addictions.

## 6. Conclusions

The rapid appearance and development of Internet addiction in adolescents is associated with the fast increase in the Internet-content spectrum within the context of the universal availability of mobile access to the Internet. These issues require urgent action to find effective treatment and means of prevention. The presence of a genetic component in IA formation is suggested by twin studies exemplified by studying different populations. However, up to the present moment, the genes involved in the mechanisms of such inheritance have not yet been identified. The analysis of the ethnic geographical distinctions of Internet addiction, with simultaneous investigations in terms of the ethnic peculiarities of the prevalence of genotypic characteristics of the populations, is considered vital. If the specialists of different spheres of expertise collaborate (e.g., pediatricians, psychologists, psychiatrists, neurologists, neurobiologists, and geneticists), then new pathophysiological mechanisms of IA formation could soon be discovered. The findings of such research may lead to the discovery of new perspectives with regards to the assessment of the fundamental neurobiological causes of the formation of Internet addiction and the personalization of a therapeutic strategy for Internet-addicted adolescents.
